# Perianal eccrine adenocarcinoma

**DOI:** 10.1186/1477-7819-5-100

**Published:** 2007-09-07

**Authors:** Tsz-Ho Leung, Henry Hsin-Chung Lee, Shih-Chang Chang

**Affiliations:** 1 Section of General Surgery, Department of Surgery, Cathay General Hospital, Taipei, Taiwan; 2 Section of Colorectal Surgery, Department of Surgery, Cathay General Hospital, Taipei, Taiwan; 3 School of Medicine, Fu Jen Catholic University, Taipei, Taiwan

## Abstract

**Background:**

Eccrine carcinoma is a quite rare malignant tumor that typically arises from a normal sweat gland and that features a rather high recurrence rate subsequent to simple excision. Given its rather poor response to adjuvant therapy, wide excision of the lesion with tumor-free margins may offer a reasonable chance for long-term control of this neoplasm.

**Case presentation:**

Herein, we report on an unusual case of perianal eccrine carcinoma, initially presenting as a perianal abscess.

**Conclusion:**

Even though eccrine carcinomas would appear to be rare, when dealing with recurrent skin tumors or recurrent perianal fistulas, the possibility of eccrine carcinoma should be considered by consulting clinicians.

## Background

There are three types of sweat glands that occur over the skin surface: eccrine, apocrine and apoeccrine. Eccrine sweat glands are one of the major sweat glands found in humans. Such sweat glands are distributed over the entire body skin surface, with the greatest density being found on the palms of the hands and soles of the feet [1]. Eccrine sweat glands are innervated by postganglionic sympathetic fibers, and their major function is thermoregulation via evaporative heat loss and electrolyte balance [1]. The majority of perianal sweat glands are of the apocrine type [1].

Eccrine carcinoma is a rare, malignant sweat gland tumor, generally featuring a slow growth rate and a rather high local recurrence rate [2]. Sweat gland carcinomas were first reported by Stout and Cooley in 1951 with the observation that, "sweat gland carcinoma often developed at the site of pre-existing nodules of longstanding and they have a propensity to metastasize" [2]. The first large study pertaining to the classification of sweat gland carcinomas was published in 1968 by Berg and McDivitt [3]. More recent attempts at classification of such carcinomas would appear to provide somewhat different results, with the relative appropriateness of such classification schemes remaining quite controversial at the time of writing [1]. Appropriate sweat gland carcinoma classification is clearly important since each of the specific sweat gland carcinomas features its own characteristic biological behavior [4]. Thus, appropriate classification of such sweat glands, and associated tumors, enables us to devise a specific and appropriate sweat gland tumor therapeutical plan, and also an associated precise prognosis, i.e. apocrine carcinomas are able to metastasize to regional lymph nodes and do so frequently, whereas eccrine carcinomas are typically not able to metastasize [4]. The incidence of sweat gland carcinoma appears to be female predominant and usually involves individuals of middle to old age [5]. Such a tumor is able to develop anywhere on the trunk of the body or on the extremities, even the eyelid [6] and nipple-areolar complex [7]. Unfortunately, as far as we are aware, sweat gland carcinomas are often under diagnosed or misdiagnosed due to their clinical rarity [8]. The clinical manifestation of eccrine carcinoma is destructive with a propensity for local recurrence [4,8]. Lymph node involvement is typically rare for eccrine carcinoma but distal metastasis is not uncommon [4].

## Case presentation

A-57-year-old female patient presented at our institution with an apparently slow growing and painful perianal mass, which had produced some serous discharge for the preceding several years. The woman denied any history of constipation or anal bleeding, and she also denied any history of major disease including malignancy. Digital examination revealed a huge (5 × 5 × 5 cm), well-defined solid tumor associated with a skin ulcer, which was located over the left posterior region of the buttock adjacent to the anus. Tracing back her surgical history, this patient had undergone perianal skin tumor excision twice previously, once in 1981 and again in 1992. On both occasions, this was due to the presence of a painful perianal mass.

**Figure 1:**
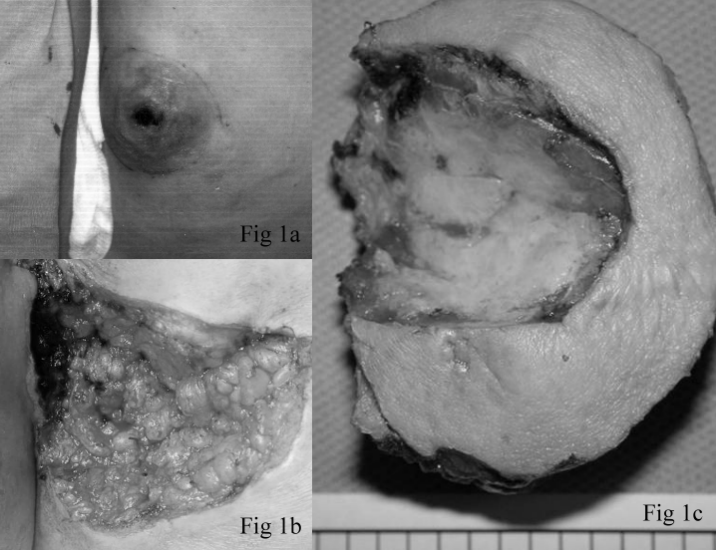
Clinicla photograph 1a) The perianal mass prior to excision was firm and revealed skin ulceration. 1b) The wound following en-bloc excision of the tumor. 1c) The excised specimen measured 7 × 6.5 × 5 cm and revealed a previous biopsy cavity. On serial sectioning, the tumor appeared well-defined, firm and gray-to-white in color.

According to her description of the previous perianal masses, they were firm and slow growing, and featured associated skin ulceration (Figure 1A). The pathology report for the mass removed in 1992 at our hospital stated it was a 5 × 5 × 3 cm perianal syringocystadenoma papilliferum. During the surgical procedure conducted in 1992, the surgical wound was repaired with a rotation skin flap as there was a rather large skin defect. She was free of disease between the two surgeries for about 8 years.

Laboratory data, including complete blood count and relevant blood biochemistry, were unremarkable. Under the initial impression of a perianal fistula or perianal skin tumor, incision biopsy was performed. The biopsy specimen was sent for frozen section examination, which revealed malignant neoplasm with myoepithelial-like proliferation. Following this histopathology report, en-bloc excision was undertaken in order to excise the tumor with a gross margin of 2 cm (Figure 1B). The post-surgery wound was left to heal openly without resorting to flap closure (Figure 1C).

**Figure 2:**
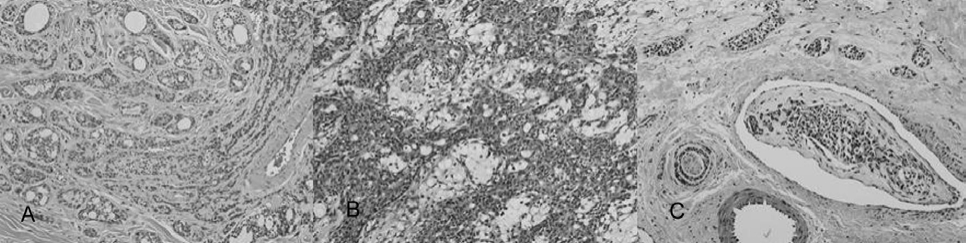
photomicrograph: 2A)Microscopic examination revealed hyperchromatic neoplastic cells arranged in solid nests, and anastomosing trabeculae with ductal formation. (H&E × 200) 2B) Microscopic examination also revealed irregular cribriform glandular structures (featuring an adenoid cystic carcinoma-like pattern) with irregular infiltrative tumor margins. (H&E × 200) 2C) Perineural invasion and lymphovascular space permeation were noted upon microscopic examination. (H&E × 200).

Subsequent to surgery, metastatic work-ups revealed multiple nodules over both of the patient's lungs, presumably secondary to the primary malignant neoplasm. Other examinations, including immunological tumor markers (CA 19-9: 16.5; CEA: 0.2; CA 125: 15.4; SCC: 0.2), sputum cytology and gynecological ultrasonography, were performed and all produced unremarkable results. The final pathology report suggested the presence of an eccrine adenocarcinoma. Microscopically, lesion sections revealed hyperchromatic neoplastic cells arranged in solid nests, anastomosing trabeculae with ductular formation (Figure 2A), a syringoid tubular or cord-like pattern, and irregular cribriform glandular structures (revealing an adenoid cystic carcinoma-like pattern) with irregular infiltrative tumor margins (Figure 2B).

The tumor cells were characterized by the presence of mild to moderate nuclear pleomorphisms, occasional distinct nucleoli, not uncommon mitotic activity, and a variable amount of eosinophilic, basophilic or clear cytoplasm featuring focal tumor necrosis. Perineural invasion and lymphovascular space permeation were also noted (Figure 2C). Immunohistochemically, the proliferated stromal cells and peripheral cells of the tumor nest or glandular structures were focally positive for SMA. The luminal tumor cells and central cells of the tumor nests were variably positive for polyclonal CEA and CK7. All of the neoplastic cells were negative for EMA, CDX2 and TTF-1, and no evident tumor cell mucin production was seen on mucicarmine and DPAS staining. The cancer invaded the dermis and subcutis. The epidermis was spared and free of pagetoid involvement.

## Discussion

Surgery is the first choice for management of such eccrine carcinomas since this type of carcinoma typically features a poor response to chemotherapy and/or radiotherapy [9]. However, it has been reported that one patient with distal metastasis secondary to an eccrine tumor responded well to tamoxifen [10]. Wide, deep surgical excision of the tumor, with verification of tumor-free margins, should offer a reasonable chance of long-term control of eccrine carcinoma [10]. The use of Mohs micrographic surgery was proposed by Wildemore et al. recently in order to attempt to decrease the recurrence rate of this type of tumor when compared to conventional surgical excision practices [11]. However, longer follow-up intervals are reportedly necessary.

For our patient, when dealing with the current tumor, we intended to review the histopathology slides prepared in 1992 from the patient's previous episode in order to compare presentations and determine whether any mistake may have been made previously regards microscopic interpretation of the prepared tissue slides.

Unfortunately, the relevant slides had been lost during transportation several years previously and we were denied such an opportunity. Following our review of the literature, as far as we were able to determine, there is one case report about a patient whose eccrine porocarcinoma could possibly have arisen from a long-standing benign eccrine poroma [13]. Otherwise, no evidence suggesting the transformation of a benign eccrine tumor to a malignant lesion has ever been found.

As for the suspicious nodules present on this patient's chest X-ray, we suggested thoracoscopic biopsy as being appropriate and, subsequent to metastasis having been confirmed, concurrent chemo-radiotherapy. However, this was refused by the patient.

## Conclusion

Even though eccrine carcinoma is a clinical rarity, when dealing with recurrent skin tumors or recurrent perianal fistulas, the possibility of eccrine carcinoma should be considered by consulting clinicians. Such a tumor can be slow growing but does feature the potential for recurrence, as well as distal metastasis [12]. A high index of suspicion and the taking of perisurgical frozen sections will likely elicit a greater diagnostic rate for this malady, and initial wide excision of the tumor with adequate margins should thus significantly reduce the likelihood of a secondary perianal procedure being necessary. Distal metastasis work-up should be performed routinely for patients afflicted with eccrine carcinoma. Although prognosis for eccrine carcinoma is somewhat guarded, repeated wide excision of the primary lesion may be necessary

## Competing interests

The author(s) declare that they have no competing interests.

## Authors' contributions

THL: Primary author, preparation and submission of manuscript

HHCL: The attending surgeon of this case and critically for important intellectual content

SCC: Contributing authors and carried out the surgical procedures with HHCL

All authors have read the final manuscript and agree to its publication.
